# (1*E*,2*E*)-2-Methyl-3-phenyl­acryl­aldehyde thio­semicarbazone

**DOI:** 10.1107/S1600536812022386

**Published:** 2012-05-23

**Authors:** Rafael Mendoza-Meroño, Santiago García-Granda

**Affiliations:** a Departamento de Química Física y Analítica, Facultad de Química, Universidad de Oviedo – CINN, C/ Julián Clavería, 8, 33006 Oviedo, Spain

## Abstract

In the crystal structure of the title compound, C_11_H_13_N_3_S, mol­ecules form centrosymmetric synthons with an *R*_2_^2^(8) graph-set motif, linked by pairs of N—H⋯S hydrogen bonds. The synthons are connected through further N—H⋯S hydrogen bonds, extending the packing to form a two-dimensional network lying parallel to (001). In addition, C—H⋯π inter­actions are observed.

## Related literature

For related compounds and their biological activity, see: Abid *et al.* (2008 ▶); Finkielsztein *et al.* (2008 ▶). For hydrogen bonding in thio­semicarbazones, see: Lima *et al.* (2002 ▶); Allen *et al.* (1997 ▶). For the use of resonance-induced hydrogen bonding in supra­molecular chemistry, see: Kearney *et al.* (1998 ▶). For hydrogen-bond motifs, see Bernstein *et al.* (1995 ▶). For a description of the Cambridge Structural Database, see: Allen (2002 ▶).
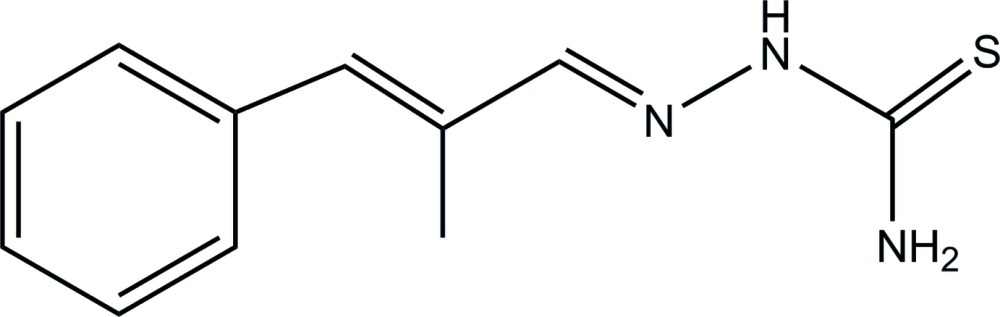


## Experimental

### 

#### Crystal data

C_11_H_13_N_3_S
*M* *_r_* = 219.30
Orthorhombic, 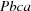

*a* = 10.9165 (5) Å
*b* = 7.8150 (3) Å
*c* = 28.0390 (14) Å
*V* = 2392.08 (19) Å^3^
*Z* = 8
Cu *K*α radiation
μ = 2.17 mm^−1^
*T* = 293 K
0.51 × 0.06 × 0.04 mm

#### Data collection

Oxford Diffraction Xcalibur Ruby Gemini diffractometer
Absorption correction: multi-scan (*CrysAlis RED*; Oxford Diffraction, 2010 ▶) *T*_min_ = 0.763, *T*_max_ = 1.000
6973 measured reflections
2241 independent reflections
1644 reflections with *I* > 2σ(*I*)
*R*_int_ = 0.037

#### Refinement

*R*[*F* ^2^ > 2σ(*F* ^2^)] = 0.039
*wR*(*F* ^2^) = 0.110
*S* = 1.04
2241 reflections
188 parameters
All H-atom parameters refined
Δρ_max_ = 0.20 e Å^−3^
Δρ_min_ = −0.15 e Å^−3^

### 

Data collection: *CrysAlis CCD* (Oxford Diffraction, 2010 ▶); cell refinement: *CrysAlis CCD*; data reduction: *CrysAlis RED* (Oxford Diffraction, 2010 ▶); program(s) used to solve structure: *SIR92* (Altomare *et al.*, 1994 ▶); program(s) used to refine structure: *SHELXL97* (Sheldrick, 2008 ▶); molecular graphics: *ORTEP-3 for Windows* (Farrugia, 1997 ▶) and *Mercury* (Macrae *et al.*, 2008 ▶); software used to prepare material for publication: *WinGX* (Farrugia, 1999 ▶), *PLATON* (Spek, 2009 ▶), *PARST95* (Nardelli, 1995 ▶) and *publCIF* (Westrip, 2010 ▶).

## Supplementary Material

Crystal structure: contains datablock(s) global, I. DOI: 10.1107/S1600536812022386/ff2068sup1.cif

Structure factors: contains datablock(s) I. DOI: 10.1107/S1600536812022386/ff2068Isup2.hkl

Supplementary material file. DOI: 10.1107/S1600536812022386/ff2068Isup3.cml

Additional supplementary materials:  crystallographic information; 3D view; checkCIF report

## Figures and Tables

**Table 1:** Hydrogen-bond geometry (Å, °) *Cg*1 is the centroid of the C1–C6 ring.

*D*—H⋯*A*	*D*—H	H⋯*A*	*D*⋯*A*	*D*—H⋯*A*
N2—H11⋯S1^i^	0.89 (2)	2.49 (3)	3.359 (2)	167 (2)
N3—H12*B*⋯S1^ii^	0.85 (3)	2.55 (3)	3.386 (2)	169 (2)
C3—H3⋯*Cg*1^iii^	0.96 (3)	2.89 (3)	3.812 (3)	161 (3)

Symmetry codes: (i) 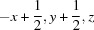; (ii) 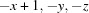; (iii) 
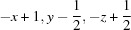
.

**Fig. 1.**
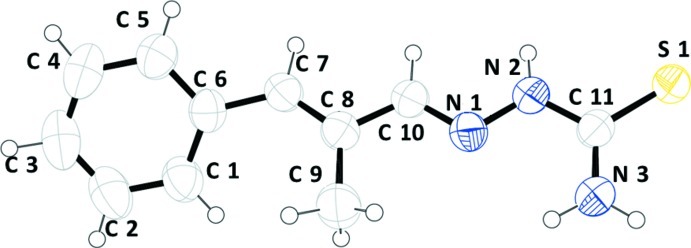
A view of the molecular structure of the title molecule showing the atom-numbering scheme. Displacement ellipsoids are drawn at the 50% probability level.

**Fig. 2.**
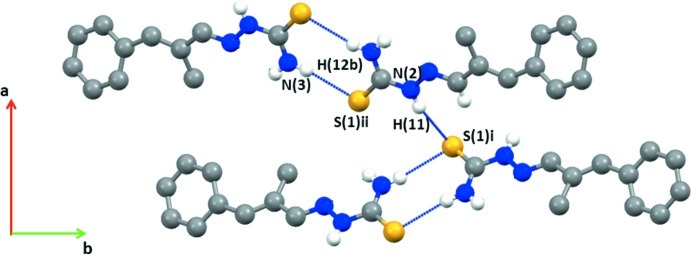
Principal hydrogen bonds. H atoms not involved in hydrogen bonding have been omitted for clarity. [Symmetry codes: (i) -x+1/2, y+1/2, z; (ii) -x+1, -y, -z]

**Fig. 3.**
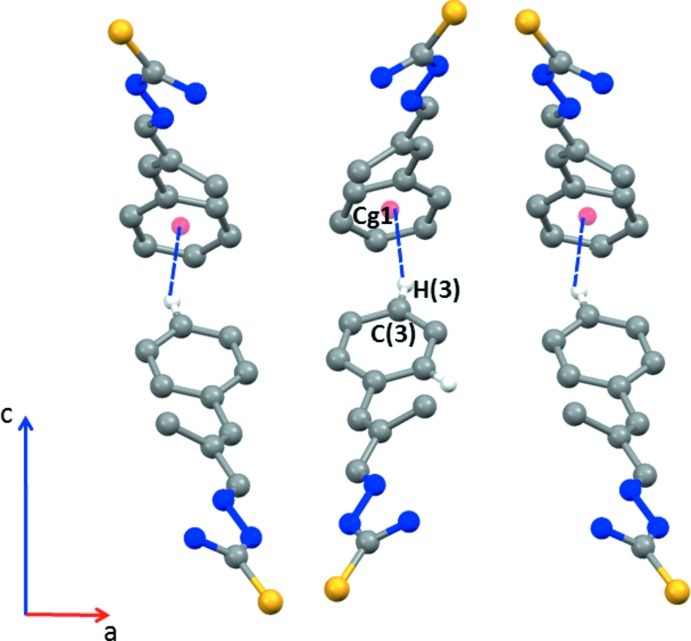
View of C—H···π interactions.

## supplementary crystallographic information

### Comment

Thiosemicarbazones have been extensively studied due to their wide range of pharmacological activities, such as antituberculosis (Abid *et al.*, 2008) and antiviral (Finkielsztein *et al.*, 2008) activities. In this work we have synthesized and crystallized a new thiosemicarbazone (I). (Fig. 1)

The values of distances N(1)–N(2) length (1.381 (2) Å.) and the dihedral angle C(10)═N(1)—N(2)—C(11) (176.32 (2) °) are similar to those found in CSD (Allen, 2002) for thiosemicarbazone systems [selected 371 hits, distance mean N—N is 1.374 Å and dihedral angle mean is 178.21 °]. According to this value —NH—C(S)—NH—N= moiety is planar and form a delocalized system, which is typical for this type of structures.

In general the molecular crystals of thiosemicarbazones are formed by hydrogen bonds interaction through –NH-C(S)-NH-N= fragment, forming in many cases *synthons*.(Lima *et al.*, 2002). Even though that C=S···H—N hydrogen bond is weaker than its C=O···H—N analogue, the effective electronegativity of S is increased by conjugative interactions between C=S and the lone pair of one or more N substituents this effect is called *resonance-induced hydrogen bonding at sulfur acceptor* (Allen *et al.* 1997). This properties have been widely exploited in supramolecular chemistry, where it has been used as a building block for anion receptors (Kearney *et al.*, 1998).

Centrosymmetric synthons (R^2^_2_(8)) (Bernstein *et al.*, 1995) are connected through N—H···S hydrogen bond to extend packing along the *a* axis (Fig 2). Intermolecular C—H···π interactions are also present in the crystal and contribute to stabilize the packing along *c* axis. (Fig.3).

### Experimental

A solution of trans-alfa-methylzimaldehyde (1.4619 g, 0.01 mol) and thiosemicarbazide (0.9144 g 0.01 mol) in absolute methanol (50 ml) was refluxed for 3 h in the presence of *p*-toluenesulfonic acid as catalyst, with continuous stirring. On cooling to room temperature the precipitate was filtered off, washed with copious cold methanol and dried in air. White single crystals of compound (I) were obtained after recrystallization from a solution in methanol.

### Refinement

All H atoms located at the difference Fourier maps and isotropically refined. At the end of the refinement the highest peak in the electron density was 0.199 eÅ ^-3^, while the deepest hole was -0.153 eÅ ^-3^.

### Crystal data

**Table:**

C_11_H_13_N_3_S	*F*(000) = 928
*M**_r_* = 219.30	*D*_x_ = 1.218 Mg m^−^^3^
Orthorhombic, *P**b**c**a*	Cu *K*α radiation, λ = 1.54180 Å
Hall symbol: -P 2ac 2ab	Cell parameters from 1905 reflections
*a* = 10.9165 (5) Å	θ = 4.1–70.4°
*b* = 7.8150 (3) Å	µ = 2.17 mm^−^^1^
*c* = 28.0390 (14) Å	*T* = 293 K
*V* = 2392.08 (19) Å^3^	Plates, white
*Z* = 8	0.51 × 0.06 × 0.04 mm

### Data collection

**Table:**

Oxford Diffraction Xcalibur Ruby Gemini diffractometer	2241 independent reflections
Radiation source: Enhance (Cu) X-ray Source	1644 reflections with *I* > 2σ(*I*)
Graphite monochromator	*R*_int_ = 0.037
Detector resolution: 10.2673 pixels mm^-1^	θ_max_ = 70.5°, θ_min_ = 5.1°
ω scans	*h* = −9→13
Absorption correction: multi-scan (*CrysAlis RED*; Oxford Diffraction, 2010)	*k* = −9→8
*T*_min_ = 0.763, *T*_max_ = 1.000	*l* = −25→34
6973 measured reflections	

### Refinement

**Table:**

Refinement on *F*^2^	Primary atom site location: structure-invariant direct methods
Least-squares matrix: full	Secondary atom site location: difference Fourier map
*R*[*F*^2^ > 2σ(*F*^2^)] = 0.039	Hydrogen site location: inferred from neighbouring sites
*wR*(*F*^2^) = 0.110	All H-atom parameters refined
*S* = 1.04	*w* = 1/[σ^2^(*F*_o_^2^) + (0.0544*P*)^2^ + 0.029*P*] where *P* = (*F*_o_^2^ + 2*F*_c_^2^)/3
2241 reflections	(Δ/σ)_max_ < 0.001
188 parameters	Δρ_max_ = 0.20 e Å^−^^3^
0 restraints	Δρ_min_ = −0.15 e Å^−^^3^

### Special details

**Table:**

Experimental. Absorption correction: CrysAlis RED (Oxford Diffraction, 2010), Empirical absorption correction using spherical harmonics, implemented in SCALE3 ABSPACK scaling algorithm.
Geometry. All esds (except the esd in the dihedral angle between two l.s. planes) are estimated using the full covariance matrix. The cell esds are taken into account individually in the estimation of esds in distances, angles and torsion angles; correlations between esds in cell parameters are only used when they are defined by crystal symmetry. An approximate (isotropic) treatment of cell esds is used for estimating esds involving l.s. planes.
Refinement. Refinement of F^2^ against ALL reflections. The weighted R-factor wR and goodness of fit S are based on F^2^, conventional R-factors R are based on F, with F set to zero for negative F^2^. The threshold expression of F^2^ > 2sigma(F^2^) is used only for calculating R-factors(gt) etc. and is not relevant to the choice of reflections for refinement. R-factors based on F^2^ are statistically about twice as large as those based on F, and R- factors based on ALL data will be even larger.

### Fractional atomic coordinates and isotropic or equivalent isotropic displacement parameters (Å^2^)

**Table:**

	*x*	*y*	*z*	*U*_iso_*/*U*_eq_	
S1	0.33592 (5)	0.16055 (7)	0.01929 (2)	0.0596 (2)	
N2	0.37558 (17)	0.4229 (2)	−0.03717 (7)	0.0586 (5)	
N1	0.44022 (15)	0.5101 (2)	−0.07187 (6)	0.0561 (4)	
C11	0.41598 (18)	0.2686 (2)	−0.02261 (7)	0.0511 (5)	
C7	0.4125 (2)	0.9191 (3)	−0.12626 (8)	0.0584 (5)	
N3	0.51732 (19)	0.2120 (3)	−0.04238 (9)	0.0692 (6)	
C10	0.4010 (2)	0.6596 (3)	−0.08224 (8)	0.0564 (5)	
C8	0.46045 (18)	0.7642 (3)	−0.11791 (7)	0.0541 (5)	
C6	0.4535 (2)	1.0540 (3)	−0.15896 (7)	0.0573 (5)	
C5	0.3674 (3)	1.1629 (3)	−0.17910 (10)	0.0713 (6)	
C1	0.5758 (2)	1.0833 (4)	−0.17014 (10)	0.0727 (7)	
C9	0.5700 (3)	0.6891 (4)	−0.14221 (11)	0.0764 (7)	
C4	0.4017 (3)	1.2909 (4)	−0.21045 (11)	0.0848 (8)	
C3	0.5221 (3)	1.3141 (4)	−0.22186 (11)	0.0890 (9)	
C2	0.6085 (3)	1.2116 (4)	−0.20136 (11)	0.0883 (9)	
H11	0.311 (2)	0.471 (3)	−0.0232 (8)	0.067 (7)*	
H12A	0.557 (2)	0.274 (3)	−0.0641 (10)	0.082 (8)*	
H7	0.339 (2)	0.944 (3)	−0.1080 (9)	0.069 (7)*	
H10	0.332 (2)	0.702 (3)	−0.0649 (8)	0.059 (6)*	
H9C	0.641 (3)	0.715 (4)	−0.1228 (13)	0.114 (12)*	
H5	0.285 (2)	1.146 (3)	−0.1724 (9)	0.081 (8)*	
H9B	0.581 (2)	0.740 (3)	−0.1745 (11)	0.089 (8)*	
H9A	0.562 (3)	0.579 (5)	−0.1461 (12)	0.125 (13)*	
H1	0.635 (2)	1.014 (3)	−0.1560 (9)	0.076 (8)*	
H2	0.690 (3)	1.220 (4)	−0.2086 (11)	0.100 (10)*	
H12B	0.546 (3)	0.116 (4)	−0.0339 (9)	0.080 (8)*	
H4	0.341 (2)	1.358 (4)	−0.2224 (10)	0.090 (9)*	
H3	0.541 (3)	1.403 (4)	−0.2441 (11)	0.098 (9)*	

### Atomic displacement parameters (Å^2^)

**Table:**

	*U*^11^	*U*^22^	*U*^33^	*U*^12^	*U*^13^	*U*^23^
S1	0.0531 (3)	0.0536 (3)	0.0720 (4)	−0.0009 (2)	−0.0050 (3)	0.0083 (3)
N2	0.0521 (10)	0.0481 (9)	0.0756 (12)	0.0041 (8)	0.0070 (9)	0.0064 (9)
N1	0.0550 (9)	0.0481 (9)	0.0652 (10)	−0.0033 (8)	0.0018 (9)	0.0002 (8)
C11	0.0478 (10)	0.0436 (10)	0.0621 (12)	−0.0031 (8)	−0.0106 (10)	−0.0031 (9)
C7	0.0551 (12)	0.0570 (12)	0.0630 (13)	−0.0016 (10)	0.0047 (11)	0.0018 (10)
N3	0.0636 (12)	0.0543 (11)	0.0898 (15)	0.0106 (10)	0.0142 (11)	0.0096 (11)
C10	0.0531 (11)	0.0504 (11)	0.0656 (13)	0.0009 (10)	0.0041 (10)	0.0010 (10)
C8	0.0543 (11)	0.0526 (11)	0.0553 (11)	−0.0041 (9)	−0.0007 (9)	−0.0026 (10)
C6	0.0696 (13)	0.0494 (11)	0.0528 (11)	−0.0034 (10)	−0.0004 (10)	−0.0014 (9)
C5	0.0783 (16)	0.0580 (13)	0.0776 (16)	0.0026 (13)	−0.0002 (13)	0.0036 (12)
C1	0.0691 (15)	0.0744 (16)	0.0744 (15)	−0.0148 (13)	−0.0067 (13)	0.0121 (13)
C9	0.088 (2)	0.0678 (17)	0.0732 (17)	0.0101 (15)	0.0213 (16)	−0.0001 (14)
C4	0.109 (2)	0.0639 (15)	0.0818 (18)	0.0019 (16)	−0.0199 (18)	0.0136 (14)
C3	0.123 (3)	0.0752 (17)	0.0687 (16)	−0.0288 (18)	−0.0133 (17)	0.0170 (14)
C2	0.088 (2)	0.095 (2)	0.0817 (18)	−0.0314 (17)	0.0004 (17)	0.0151 (16)

### Geometric parameters (Å, º)

**Table:**

S1—C11	1.690 (2)	C6—C5	1.388 (3)
N2—C11	1.347 (3)	C6—C1	1.390 (3)
N2—N1	1.381 (2)	C5—C4	1.384 (4)
N2—H11	0.89 (2)	C5—H5	0.93 (3)
N1—C10	1.278 (3)	C1—C2	1.378 (4)
C11—N3	1.314 (3)	C1—H1	0.93 (3)
C7—C8	1.339 (3)	C9—H9C	0.97 (3)
C7—C6	1.467 (3)	C9—H9B	1.00 (3)
C7—H7	0.97 (2)	C9—H9A	0.87 (4)
N3—H12A	0.89 (3)	C4—C3	1.364 (5)
N3—H12B	0.85 (3)	C4—H4	0.91 (3)
C10—C8	1.446 (3)	C3—C2	1.365 (4)
C10—H10	0.96 (2)	C3—H3	0.96 (3)
C8—C9	1.497 (3)	C2—H2	0.92 (3)
			
C11—N2—N1	119.17 (18)	C4—C5—C6	121.2 (3)
C11—N2—H11	120.1 (15)	C4—C5—H5	119.7 (17)
N1—N2—H11	120.5 (15)	C6—C5—H5	119.1 (16)
C10—N1—N2	116.09 (17)	C2—C1—C6	120.8 (3)
N3—C11—N2	116.7 (2)	C2—C1—H1	120.9 (16)
N3—C11—S1	124.07 (17)	C6—C1—H1	118.3 (16)
N2—C11—S1	119.25 (16)	C8—C9—H9C	108 (2)
C8—C7—C6	129.7 (2)	C8—C9—H9B	110.5 (16)
C8—C7—H7	114.3 (14)	H9C—C9—H9B	109 (3)
C6—C7—H7	116.0 (14)	C8—C9—H9A	111 (2)
C11—N3—H12A	121.2 (17)	H9C—C9—H9A	111 (3)
C11—N3—H12B	119.2 (18)	H9B—C9—H9A	107 (3)
H12A—N3—H12B	120 (3)	C3—C4—C5	120.4 (3)
N1—C10—C8	121.6 (2)	C3—C4—H4	122.7 (18)
N1—C10—H10	117.6 (13)	C5—C4—H4	116.9 (18)
C8—C10—H10	120.7 (13)	C2—C3—C4	119.3 (3)
C7—C8—C10	117.15 (19)	C2—C3—H3	123.5 (18)
C7—C8—C9	126.0 (2)	C4—C3—H3	117.3 (18)
C10—C8—C9	116.9 (2)	C3—C2—C1	121.1 (3)
C5—C6—C1	117.2 (2)	C3—C2—H2	122.4 (19)
C5—C6—C7	119.2 (2)	C1—C2—H2	116 (2)
C1—C6—C7	123.5 (2)		
			
C11—N2—N1—C10	−176.32 (19)	C8—C7—C6—C5	149.4 (2)
N1—N2—C11—N3	1.0 (3)	C8—C7—C6—C1	−32.5 (4)
N1—N2—C11—N3	1.0 (3)	C1—C6—C5—C4	2.4 (4)
N1—N2—C11—S1	−179.15 (14)	C7—C6—C5—C4	−179.4 (2)
N1—N2—C11—S1	−179.15 (14)	C5—C6—C1—C2	−2.3 (4)
N2—N1—C10—C8	179.95 (18)	C7—C6—C1—C2	179.6 (2)
C6—C7—C8—C10	178.3 (2)	C6—C5—C4—C3	−0.8 (4)
C6—C7—C8—C9	−1.8 (4)	C5—C4—C3—C2	−1.1 (5)
N1—C10—C8—C7	180.0 (2)	C4—C3—C2—C1	1.3 (5)
N1—C10—C8—C9	0.1 (3)	C6—C1—C2—C3	0.5 (5)

### Hydrogen-bond geometry (Å, º)

Cg1 is the centroid of the C1–C6 ring.

**Table:**

*D*—H···*A*	*D*—H	H···*A*	*D*···*A*	*D*—H···*A*
N2—H11···S1^i^	0.89 (2)	2.49 (3)	3.359 (2)	167 (2)
N3—H12*B*···S1^ii^	0.85 (3)	2.55 (3)	3.386 (2)	169 (2)
C3—H3···*Cg*1^iii^	0.96 (3)	2.89 (3)	3.812 (3)	161 (3)

Symmetry codes: (i) −*x*+1/2, *y*+1/2, *z*; (ii) −*x*+1, −*y*, −*z*; (iii) −*x*+1, *y*−1/2, −*z*+1/2.
